# Supplementary figures and images for: Struo2: efficient metagenome profiling database construction for ever-expanding microbial genome datasets
Source: PeerJ. 2021 Sep 16;9:e12198. doi: 10.7717/peerj.12198 (PMC8450008; doi:10.7717/peerj.12198)

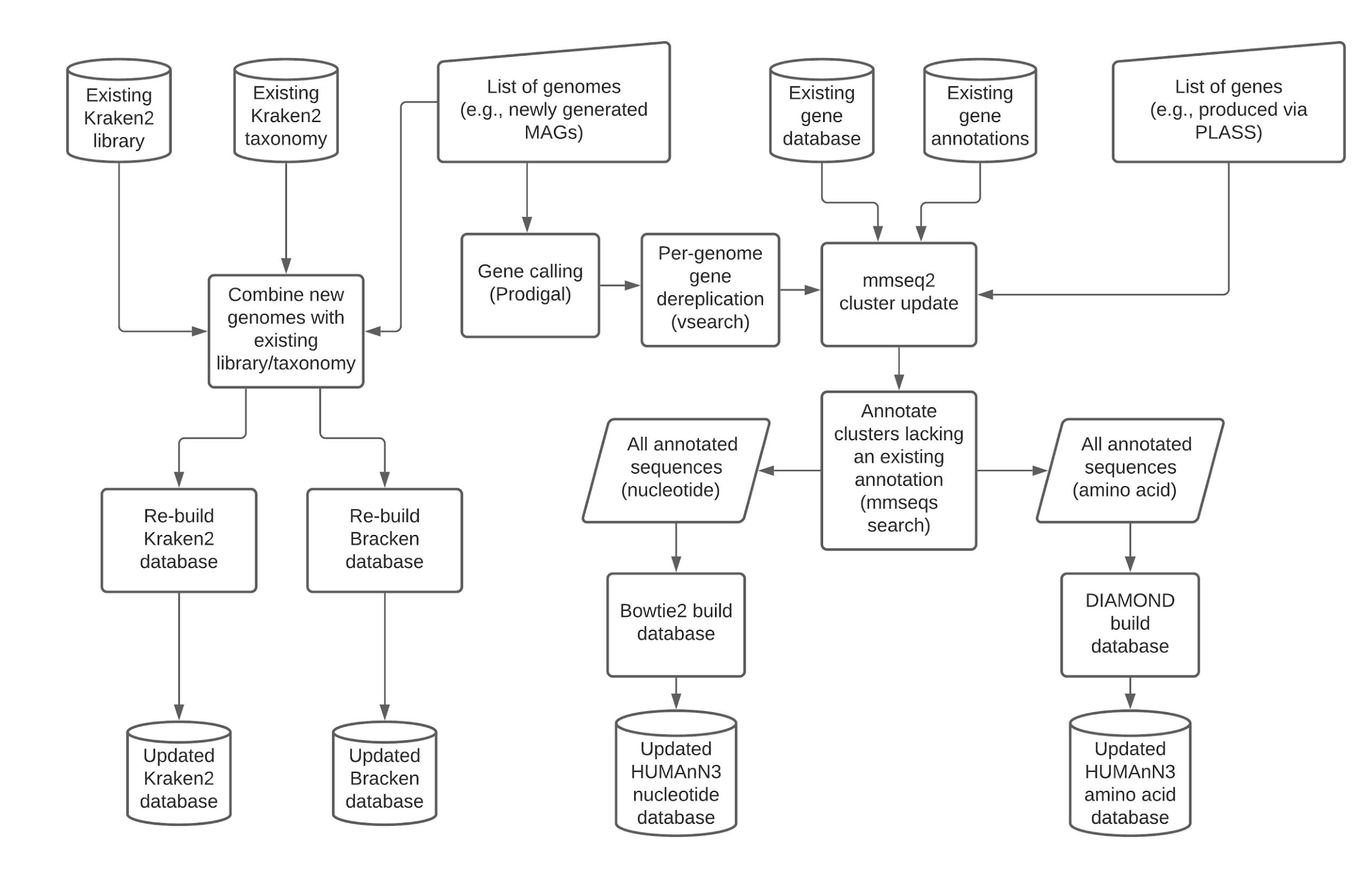

Supplement: Supplemental Information 1 — Cylinders are input or output files, squares are processes, and right-tilted rhomboids are intermediate files. Existing Kraken2, Bracken, genes, and HUMAnN3 databases can be updated with new genomes, while only existing genes and HUMAnN3 databases can be updated with new individual gene sequences. [file peerj-09-12198-s001.png]

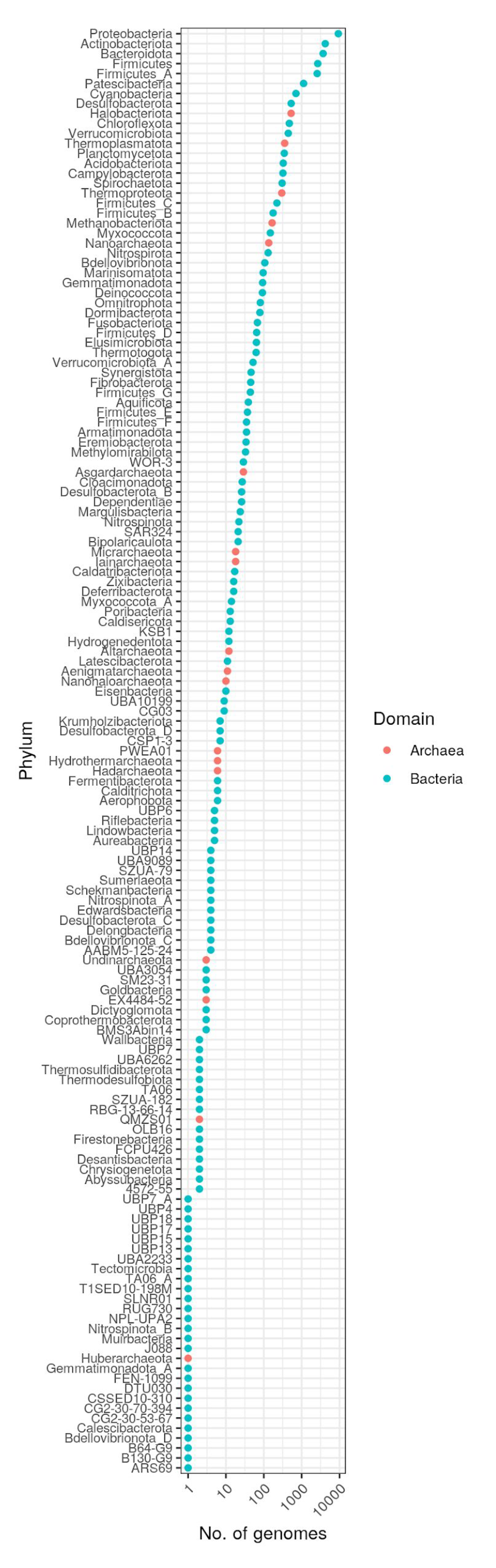

Supplement: Supplemental Information 2 — See the Materials & Methods for information on how genomes were selected. The phylum names shown are based on the GTDB taxonomy. [file peerj-09-12198-s002.png]
